# Tooth demineralization and associated factors in patients on fixed orthodontic treatment

**DOI:** 10.1038/srep36383

**Published:** 2016-11-02

**Authors:** Elías Nahúm Salmerón-Valdés, Edith Lara-Carrillo, Carlo Eduardo Medina-Solís, Norma Leticia Robles-Bermeo, Rogelio José Scougall-Vilchis, Juan Fernando Casanova-Rosado, América Patricia Pontigo-Loyola, Miguel Ángel Fernández Barrera

**Affiliations:** 1 Advanced Studies and Research Centre in Dentistry “Dr. Keisaburo Miyata” of School of Dentistry at Autonomous University State of Mexico, Toluca, México; 2 Academic Area of Dentistry of Health Sciences Institute at Autonomous University of Hidalgo State, Pachuca, Mexico; 3 School of Dentistry of Autonomous University of Campeche, Campeche, México

## Abstract

Dental demineralization was determined in patients at three time points during fixed orthodontic treatment. A multiple cross-sectional study included 108 patients divided into three different groups: (1) beginning of orthodontic treatment; (2) one year into treatment; and (3) two years into treatment. Demineralization was estimated using a DIAGNOdent pen. We obtained data from multiple tooth-by-tooth demineralization readings combined with salivary pH and patients’ oral hygienic and dietary behaviors. A t-test for independent samples and Spearman´s correlation were performed. No demineralizations differences were found between the initial stage and one year into treatment. Between one and two years small differences were observed, but demineralization increased between the initial stage and second treatment year, predominating in upper right central incisors (p = 0.056), upper left lateral incisors (p = 0.040), both upper canines (p = 0.055 and p = 0.040, respectively) and first left premolars (p = 0.034 and p = 0.053, respectively). We did not find associations between oral hygiene and dietary behaviours or salivary pH. In conclusion, demineralization occurred in first year of treatment and increased during second year, predominating in the upper arch and the left side mainly in upper right central incisors, upper left lateral incisors, both upper canines, and first left premolars.

Orthodontic treatment is an elective procedure to improve the patient’s dentofacial appearance. A major barrier in achieving this goal is the risk to develop demineralization areas adjacent to orthodontic brackets. There is a well-established relationship between orthodontic fixed appliance use and enamel demineralization. The occurrence of enamel demineralization is a significant problem related to fixed orthodontic appliances. Demineralization varies from no perceptible change to white spots on the enamel, which is the first clinical sign of enamel demineralization, to cavitation. These areas may require further treatment after orthodontic therapy to mask or remove these spots. Many prior studies have reported that patients who use brackets have up to 85% risk of developing white spot lesions^1^. Thus, in a recent meta-analysis of 14 studies evaluated for white spot lesions, the incidence of new carious lesions formed during orthodontic treatment in patients was 45.8%, and the prevalence of lesions in patients undergoing orthodontic treatment was 68.4%^2^.

It has been shown that orthodontic treatment induces changes in the oral environment such as an increase in bacterial concentrations and alterations in pH, salivary buffering capacity, and salivary flow. Furthermore, all of these changes are closely associated with hygienic and dietary behaviours of the patient^3^,^4^.

The placement of orthodontic appliances increases retention areas, and irregular surfaces of brackets and bands provide protection to microorganisms from physical forces. Therefore, cleaning becomes more complicated, which favours plaque accumulation^3^,^5^.

Dietary behaviours significantly influence demineralization; if fermentable substances are continuously ingested, acid production by plaque exceeds neutralization by the saliva, and demineralization occurs^6^. When carbohydrate availability is excessive, pH drops and remains at this lower level^7^.

There are technologies to help in early demineralization detection; one device is DIAGNOdent^8^,^9^,^10^,^11^. The DIAGNOdent uses laser fluorescence to detect incipient caries by measuring the fluorescence of bacterial products called porphyrins. This technology does not require the presence of clinically visible carious lesions. This theory has been supported by the fact that the DIAGNOdent device does not detect lesions produced in the laboratory by acid solutions without microbiological activity. The device generates a laser light that is absorbed by the materials found in the tooth and then re-emitted as infrared fluorescence^12^. Several studies have shown that the sensitivity of DIAGNOdent ranged from 0.17 to 0.87, and the specificity ranged from 0.72 to 0.98 for detection of caries lesions^9^,^13^,^14^,^15^,^16^. The laser-optical DIAGNOdent is known to offer high sensitivity in caries detection. Aljehani *et al.* showed that the correlation between DIAGNOdent and visual examination was 0.63 and reported that DIAGNOdent was a more objective and reproducible method than visual examination for detecting and long-term follow up of incipient carious lesions^17^. Application of this device has been recommended (in addition to the clinical examination) for visually inaccessible areas and assessment of vestibular tooth surfaces around brackets. The early detection of caries lesions is important to provide proper and non-invasive management as lesions at this stage have the potential to be remineralized and can be monitored over time. Therefore, the DIAGNOdent pen appears to be a suitable tool for classifying the severity of potential surface demineralization during multibracket treatment^18^,^19^.

The aim of the present study was to determine the level of demineralization in patients during orthodontic treatment and its association with salivary pH and hygienic and dietary factors.

## Material and Methods

This was a multiple cross-sectional study in 108 patients in three different stages of orthodontic treatment. Patients were in each group (n = 36): (1) at the beginning of treatment; (2) one year into treatment; and (3) two years into treatment. We did not use any sampling approach (convenience sampling). The patients in the first stage were free of caries lesions, since those lesions are one of the prerequisites for entry into the orthodontic treatment. The study was conducted in patients from the Orthodontic Clinic at the Research Center and Advanced Studies in Dentistry of the Autonomous University of State of Mexico (UAEM). All patients or their guardians consented to participation after obtaining information about the study.

We also administered a face-to-face questionnaire in order to collect data regarding patients’ sociodemographic characteristics as well as their hygienic and dietary behaviours. This questionnaire was given to all patients and consisted of with nine closed questions: (1) gender; (2) occupation; (3) brushing time; (4) frequency of daily brushing; (5) use of dental auxiliaries; (6) frequency of changing toothbrush; (7) snacks between meals; (8) snacking time; and (9) frequency of daily snacks.

Clinical data were taken from the patient while he/she was sitting on a dental chair under artificial light with all necessary infection control barriers. Demineralization was estimated using a DIAGNOdent pen 2190 (Kavo, Warthausen, Germany). This device was previously calibrated, according to the manufacturer’s instructions. The results are shown as readings from 0 to 99 and appear on the digital display located on the back of the device. According to the manufacturer, 0–12 is considered low risk, medium is 13–24, and >25 is high for smooth surfaces; for proximal areas, 0–7 is low, medium is 8–15, and >16 is high.

We analyzed six points on each tooth consisting of four points on buccal surfaces (mesial, distal, gingival. and incisal or oclusal to the bracket) and two points on interproximal areas (mesial and distal) from premolar to premolar in both dental arches. In our study, we obtained the average of six measurements (four around the bracket and two in the interproximal areas), because orthodontic appliances consisting of brackets; elastic chains, springs, and archwires and are continuous devices that prevent proper oral hygiene by the patient. These devices prevent the toothbrush bristles from reaching between the interproximal spaces. For this reason is important to include the degree of mineralization of these areas in order to have a representative measure of the condition of the entire tooth.

The same DIAGNOdent pen was used throughout the study, and one examiner performed the measurements. The surface was dried with compressed air for 5 sec before each measurement. Each point was scanned two times with the pen, and the highest value from the two readings was registered. When all measurements were obtained, we then averaged the values to find a total value by each tooth. Subsequently, an average of demineralization values (total demineralization) by stage was obtained (at the beginning and at one and two years).

The salivary pH was calculated using test strips (Universal strips pH 0–14; Meerck KGaA, Darmstadt, Germany), which were placed under the patient’s tongue for two minutes. For pH measurements, patients were requested not to eat for two hours before taking the sample at the same time of day, just before starting their orthodontic consultation, because ingested food, as well as the consumption of a drink and/or toothbrushing, can modify the salivary pH. For the collection of these samples, all participants were requested to be as quiet as possible. The test strips were immediately compared with a sampling table to determine degree of acidity or alkalinity.

The information collected from questionnaires and clinical examination was analysed with SPSS version 19. We performed a descriptive analysis of the population by using central tendency and dispersion measurements for the quantitative variables and frequency and percentages for the qualitative variables. Subsequently, bivariate analyses were performed to determine if there was any difference using a t-test for independent samples and Spearman´s correlation. The statistical level of significance was set at 0.05.

### Ethical aspects

Written consent was obtained from all the patients/guardians. The protocol was approved by the ethics committee review board of the Research Center and Advanced Studies in Dentistry of the Autonomous University of State of Mexico. All procedures performed in studies involving human participants were in accordance with the ethical standards of the institutional and/or national research committee and with the 1964 Helsinki declaration and its later amendments or comparable ethical standards.

## Results

Table 1 shows descriptive data with respect to the three stages in this study according to the questionnaire responses. Demineralization in the upper arch showed no statistically significant differences when comparing first and second stages. Between one and two years of treatment, we found significant differences only in left first premolar (p = 0.025). When comparing the treatment beginning and two years, differences were detected in the right canine (p = 0.055), right central incisor (p = 0.056), left lateral incisor (p = 0.040), left canine (p = 0.040), and left first premolar (p = 0.034) (Table 2). Similarly in the lower arch, no statistically significant differences were found between the treatment beginning and one year of treatment. When comparing demineralization in the left first premolar between one and two years of treatment and between the treatment beginning and two years, differences were observed (p = 0.018 and p = 0.053, respectively) (Table 2). No differences in demineralization at the initial stage and the first year of orthodontic treatment were observed, however, between one year and two years in orthodontic treatment small differences were observed, but these differences increased when the initial stage was compared with the second treatment year (Table 2). Using demineralization values for each tooth, we obtained a total value of demineralization for each orthodontic stage. This average value was compared with oral hygiene and dietary behaviours; and no significant dietary or hygienic factors were found that may affect the presence of demineralization (Table 3).

Similarly, age, salivary pH, and total demineralization by each stage were correlated by Spearman’s analysis and no statistically significant differences between these parameters were found (p > 0.05) (Table 4).

## Discussion

The results of this study indicated that demineralization may be a significant problem during orthodontic treatment, increasing from the initiation of orthodontic treatment to one year and continuing to increase into the second year of treatment; a patient´s oral hygiene and dietary behaviours seemed to be irrelevant to the degree of demineralization.

Tufekci *et al.* reported that during orthodontic treatment the number of demineralization injuries increased significantly during the first six months and continued to increase up to 12 months^5^. Our results agreed with results from that study, which, however, evaluated the demineralization only for a year. In our study, however, we evaluated up to two years of treatment and found that demineralization continues during that period.

In the study groups, demineralization predominated in the upper arch on the left side. About 90% of our population is right-handed. Claydon reported that in populations with poor oral hygiene, the most significant loss of the periodontal attachment is in the maxillary arch^20^. The patterns of toothbrushing for most right-handed people begins with brushing the buccal surfaces of the anterior teeth or left side; consequently, the most severe gingival recession and abrasion defects are localized to the buccal surfaces on the left side. These lesions can add to demineralization caused by complicated brushing on the opposite side.

In this study, the teeth most affected by demineralization were the upper left central incisors, upper right lateral incisors, both upper canines, and first left premolars. The primary etiology of demineralization is bacterial acids, which may affect sites that are less protected by saliva such as the anterior teeth^20^.

Enaia *et al.* reported that the central and lateral incisors are the teeth most frequently affected^21^. Other authors such as Derks *et al.* reported that the first premolars are the teeth most affected by demineralization during orthodontic treatment. In our research, the left first premolars were presented^22^ as the teeth with the highest risk of demineralization during the last stages of treatment.

It has been reported that a majority of interproximal lesions are incipient, thus making clinical diagnosis difficult. It appears to be necessary to use other diagnostic tools in conjunction with clinical diagnosis.

Literature studies report that salivary pH increases after placement of the orthodontic appliances^4^. We did not find any published articles, however, that associated demineralization with the time, behaviours, and sociodemographic characteristics of the patients undergoing orthodontic treatment. With regard to hygienic and dietary factors associated with total demineralization that were analyzed in each study stage, we did not find any significantly different between associations. We concluded that patterns of demineralization were equal. Future longitudinal follow-up studies with larger sample sizes are recommended to eliminate circumstances that may affect the differences across study groups.

### Limitations and conclusions

The present study has some limitations, therefore its interpretation should be cautious and its design (a cross-sectional study) was affected by temporal ambiguity. The cause and effect were measured at the same time. For that reason, this study precludes confirming causal relationships but favours associations only. In conclusion, demineralization occurs in first year of treatment and increases during the second year. The results obtained in this study allowed us to identify the teeth most frequently undergoing demineralisation. Predominance of demineralization was seen in the upper arch and on the left side, mainly affecting upper right central incisor, upper left lateral incisors, both upper canines, and first left premolars. Oral hygiene habits, dietary behaviours, and salivary pH did not show any significant associations with total demineralization. Demineralization patterns were the same even when the dietary and hygienic behaviours differed. It is important to implement specific preventive measures in patients who are undergoing orthodontic treatment.

## Additional Information

**How to cite this article**: Salmerón-Valdés, E. N. *et al.* Tooth demineralization and associated factors in patients on fixed orthodontic treatment. *Sci. Rep.* **6**, 36383; doi: 10.1038/srep36383 (2016).

**Publisher’s note:** Springer Nature remains neutral with regard to jurisdictional claims in published maps and institutional affiliations.

## Figures and Tables

**Table 1:** Descriptive data of the patients (n = 36 each stage).

Numerical Variables (average (S.D.)			
Variables	Beginning	1 year	2 years
Age	16.94 (4.25)	19.50 (1.88)	19.25 (5.04)
pH	5.77 (0.68)	5.91 (1.36)	6.33 (1.01)
**Categorical Variables (in %)**			
Gender			
Male	47.2	44.4	27.8
Female	52.8	55.6	72.2
Occupation			
Student	83.3	97.2	83.3
Other (different to student)	16.7	2.8	16.7
Brushing time			
Before meals	22.2	2.8	2.8
After meals	77.8	97.2	97.2
Times patients brushed their teeth daily			
1–2	44.4	75.0	44.4
3 or more	55.6	25.0	55.6
Use of dental auxiliaries			
Yes	69.4	52.8	66.7
No	30.6	47.2	33.3
Frequency of toothbrush changes			
1–3 months	83.3	94.4	88.9
More than 3 months	16.7	5.6	11.1
Snacks between meals			
Yes	88.9	94.4	97.2
No	11.1	5.6	2.8
Snacks time			
In the morning	78.1	76.5	71.4
In the afternoon	21.9	23.5	28.6
Times patient snacks between meals			
1 – 3 times	88.9	86.1	100.0
More 3 times	11.1	13.9	0

**Table 2:** Demineralization comparison between stages.

Teeth	Beginnging	1 year	p value	1 year	2 years	p value	Beginning	2 years	p value
4	2.40 (1.22)	2.55 (2.10)	0.716	2.55 (2.10)	3.43 (3.53)	0.204	2.40 (1.22)	3.43 (3.53)	0.103
5	2.14 (0.85)	3.07 (4.11)	0.191	3.07 (4.11)	2.74 (2.15)	0.668	2.14 (0.85)	2.74 (2.15)	0.129
6	1.95 (1.21)	2.98 (6.49)	0.354	2.98 (6.49)	3.00 (2.97)	0.988	1.95 (1.21)	3.00 (2.97)	0.055*
7	2.04 (1.64)	2.74 (3.41)	0.275	2.74 (3.41)	2.75 (2.97)	0.981	2.04 (1.64)	2.75 (2.97)	.212
8	1.74 (1.21)	2.59 (2.78)	0.097	2.59 (2.78)	2.70 (2.71)	0.864	1.74 (1.21)	2.70 (2.71)	0.056*
9	1.71 (0.93)	2.94 (3.78)	0.062	2.94 (3.78)	2.52 (2.54)	0.585	1.71 (0.93)	2.52 (2.54)	0.076
10	1.56 (1.05)	3.07 (5.00)	0.080	3.07 (5.00)	2.51 (2.52)	0.550	1.56 (1.05)	2.51 (2.52)	0.040*
11	1.73 (0.94)	2.50 (4.23)	0.292	2.50 (4.23)	2.64 (2.45)	0.857	1.73 (0.94)	2.64 (2.45)	0.040*
12	2.05 (1.06)	1.94 (1.33)	0.698	1.94 (1.33)	3.37 (3.51)	0.025*	2.05 (1.06)	3.37 (3.51)	0.034*
13	2.48 (1.19)	2.90 (5.75)	0.665	2.90 (5.75)	3.12 (2.09)	0.828	2.48 (1.19)	3.12 (2.09)	0.112
20	3.52 (2.52)	3.74 (6.60)	0.857	3.74 (6.60)	4.19 (3.72)	0.721	3.52 (2.52)	4.19 (3.72)	0.377
21	2.87 (1.61)	2.40 (1.82)	0.258	2.40 (1.82)	4.78 (5.61)	0.018*	2.87 (1.61)	4.78 (5.61)	0.053*
22	2.47 (1.47)	2.60 (2.78)	0.806	2.60 (2.78)	3.83 (7.01)	0.331	2.47 (1.47)	3.83 (7.01)	0.259
23	3.30 (2.23)	4.42 (9.66)	0.500	4.42 (9.66)	3.23 (3.26)	0.485	3.30 (2.23)	3.23 (3.26)	0.911
24	3.99 (3.86)	5.75 (10.70)	0.356	5.75 (10.70)	3.16 (4.22)	0.182	3.99 (3.86)	3.16 (4.22)	0.391
25	4.06 (3.76)	7.92 (17.38)	0.196	7.92 (17.38)	3.67 (5.51)	0.166	4.06 (3.76)	3.67 (5.51)	0.728
26	3.27 (2.17)	5.71 (12.51)	0.254	5.71 (12.51)	4.28 (5.79)	0.537	3.27 (2.17)	4.28 (5.79)	0.331
27	2.32 (1.45)	4.04 (6.01)	0.103	4.04 (6.01)	3.13 (3.38)	0.442	2.32 (1.45)	3.13 (3.38)	0.189
28	2.91 (2.62)	5.18 (12.02)	0.273	5.18 (12.02)	3.11 (4.82)	0.340	2.91 (2.62)	3.11 (4.82)	0.833
29	3.15 (2.19)	5.37 (11.85)	0.273	5.37 (11.85)	3.62 (5.38)	0.420	3.15 (2.19)	3.62 (5.38)	0.635

Data showed average (S.D.).

p values based on t-test analysis for independent samples. p ≤ 0.05*.

Teeth using ADA classification.

**Table 3:** Comparison between total demineralization (DIAGNOdent values) and categorical variables.

Variables	Beginning	p value	1 year	p value	2 years	p value
Gender						
Male	2.55 (0.71)		3.51 (3.70)		3.63 (3.67)	
Female	2.61 (1.28)	0.866	3.89 (6.92)	0.843	3.16 (2.62)	0.669
Occupation						
Student	2.46 (1.03)		3.80 (5.71)		3.40 (3.15)	
Other (different to student)	3.20 (0.91)	0.113	.80 (0.00)	0.607	2.73 (0.90)	0.611
Brushing time						
Before meals	2.68 (0.91)		3.16 (0.00)		0.33 (0.00)	
After meals	2.55 (1.09)	0.759	3.74 (5.73)	0.922	3.37 (2.90)	0.308
Times patients brushed their teeth daily						
1–2	2.78 (1.12)		4.34 (6.40)		3.59 (3.94)	
3 or more	2.42 (0.97)	0.305	1.87 (1.15)	0.264	3.05 (1.76)	0.588
Use of dental auxiliaries						
Yes	2.58 (1.06)		2.12 (1.16)		3.68 (3.32)	
No	2.58 (1.04)	0.992	5.51 (7.87)	0.072	2.51 (1.67)	0.258
Frequency of toothbrush changes						
1–3 months	2.58 (1.09)		3.88 (5.78)		3.32 (3.06)	
More than 3 months	2.58 (0.80)	0.997	1.00 (0.55)	0.493	3.01 (1.05)	0.843
Snacks between meals						
Yes	2.52 (0.94)		3.76 (5.80)		3.35 (2.92)	
No	3.12 (1.76)	0.280	3.05 (2.18)	0.867	1.13 (0.00)	0.459
Snacks time						
In the morning	2.53 (0.83)		4.29 (6.55)		3.88 (3.27)	
In the afternoon	2.46 (1.32)	0.862	2.02 (1.12)	0.341	2.04 (1.00)	0.093
Times patient snacks between meals						
1 – 3 times	2.56 (1.05)		3.94 (6.06)		3.29 (2.90)	
More 3 times	2.78 (1.08)	0.690	2.35 (1.22)	0.567	NC	NC

NC = no calculated.

Data showed average (SD).

p values based on t-test analysis for independent samples.

**Table 4:** Correlations found between total demineralization by orthodontic stages, age and salivary pH.

			Age B	pH B	Total_dem B
Beginning	Age B	Correlation coefficient	1.000	−0.168	0.117
		p value	.	0.327	0.496
	pH B	Correlation coefficient	−0.168	1.000	−0.292
		p value	0.327	.	0.084
	Total_dem B	Correlation coefficient	0.117	−0.292	1.000
		p value	0.496	0.084	.
			Age 1	pH 1	Total_dem 1
1 year	Age 1	Correlation coefficient	1.000	0.068	0.222
		p value	.	0.693	0.193
	pH 1	Correlation coefficient	0.068	1.000	−0.014
		p value	0.693	.	0.936
	Total_dem 1	Correlation coefficient	0.222	−0.014	1.000
		p value	0.193	0.936	.
			Age 2	pH 2	Total_dem 2
2 years	Age 2	Correlation coefficient	1.000	0.108	0.250
		p value	.	0.529	0.141
	pH 2	Correlation coefficient	0.108	1.000	0.014
		p value	0.529	.	0.937
	Total_dem 2	Correlation coefficient	0.250	0.014	1.000
		p value	0.141	0.937	.

p values based on Spearman’s correlation coefficient.

B = beginning treatment; 1 = one year on treatment; 2 = two years on treatment.

## References

[b1] Nascimento P. L., Fernandes M. T., Figueiredo F. E. & Faria-E-Silva A. L. Fluoride-releasing materials to prevent white spot lesions around orthodontic brackets: A Systematic Review. Braz Dent J. 27, 101–107 (2016). 27007355 10.1590/0103-6440201600482

[b2] Sundararaj D., Venkatachalapathy S., Tandon A. & Pereira A. Critical evaluation of incidence and prevalence of white spot lesions during fixed orthodontic appliance treatment: A meta-analysis. J Int Soc Prev Community Dent. 5, 433–439 (2015). 26759794 10.4103/2231-0762.167719 PMC4697225

[b3] Cahuana-Vásquez R. A. *et al.* Effect of frequency of sucrose exposure on dental biofilm composition and enamel demineralization in the presence of fluoride. Caries Res. 41, 9–15 (2007). 17167254 10.1159/000096100

[b4] Lara-Carrillo E., Montiel-Bastida N. M., Sanchez-Pérez L. & Alanís-Tavira J. Changes in the oral environment during four stages of orthodontic treatment. Korean J Orthod. 40, 95–105 (2010).

[b5] Tufekci E., Dixon J. S., Gunsolley J. C. & Lindauer S. J. Prevalence of white spot lesions during orthodontic treatment with fixed appliances. Angle Orthod. 81, 206–210 (2011). 21208070 10.2319/051710-262.1 PMC8925248

[b6] Paschos E., Kurochkina N., Huth K. C., Hansson C. S. & Rudzki-Janson I. Failure rate of brackets bonded with antimicrobial and fluoride-releasing, self-etching primer and the effect on prevention of enamel demineralization. Am J Orthod Dentofacial Orthop. 135, 613–620 (2009). 19409344 10.1016/j.ajodo.2008.01.016

[b7] Wiltshire W. A. *In vitro* and *in vivo* fluoride release from orthodontic elastomeric ligature ties. Am J Orthod Dentofacial Orthop. 115, 288–292 (1999). 10066977 10.1016/s0889-5406(99)70331-8

[b8] Zandoná A. & Zero D. T. Diagnostic tools for early caries detection. J Am Dent Assoc. 137, 1675–1684 (2006). 17138712 10.14219/jada.archive.2006.0113

[b9] Kouchaji C. H. Comparison between a laser fluorescence device and visual examination in the detection of occlusal caries in children. Saudi Dent J. 24, 169–174 (2012). 23960547 10.1016/j.sdentj.2012.07.002 PMC3729294

[b10] Mendes F. M., Siqueira W. L., Mazzitelli J. F., Pinheiro S. L. & Bengtson A. L. Performance of DIAGNOdent for detection and quantification of smooth-surface caries in primary teeth. J Dent. 33, 79–84 (2005). 15652172 10.1016/j.jdent.2004.10.010

[b11] Huth K. C. *et al.* *In vivo* performance of a laser fluorescence device for the approximal detection of caries in permanent molars. J Dent. 38, 1019–1026 (2010). 20837089 10.1016/j.jdent.2010.09.001

[b12] Pretty I. A. & Maupomé G. A closer look at diagnosis in clinical dental practice part 5. Emerging technologies for caries detection and diagnosis. J Can Dent Assoc. 70, 540a–540i (2004). 15363214

[b13] Lussi A., Mergert B., Longbottom C., Reich E. & Francescut P. Clinical performance of a laser fluorescence device for detection of oclusal caries lesions. Eur J Oral Sci. 109, 14–19 (2001). 11330928 10.1034/j.1600-0722.2001.109001014.x

[b14] Tagtekin D. A. *et al.* Caries detection with DIAGNOdent and ultrasound. Oral Surg Oral Med Oral Pathol Oral Radiol Endod. 106, 729–735 (2008). 18656396 10.1016/j.tripleo.2008.05.010

[b15] Kühnisch J., Bücher K. & Hickel R. The intra/inter-examiner reproducibility of the new DIAGNOdent Pen on occlusal sites. J Dent. 35, 509–512 (2007). 17395355 10.1016/j.jdent.2007.02.001

[b16] Huth K. C. *et al.* Clinical performance of a new laser fluorescence device for detection of occlusal caries lesions in permanent molars. J Dent. 36, 1033–1040 (2008). 18930575 10.1016/j.jdent.2008.08.013

[b17] Aljehani A., Yousif M. A., Angmar-Mânsson B. & Shi X. Q. Longitudinal quantification of incipient carious lesions in postorthodontic patients using a fluorescence method. Eur J Oral Sci. 114, 430–434 (2006). 17026510 10.1111/j.1600-0722.2006.00395.x

[b18] Bechtold T. E., Sobiegalla A., Markovic M., Berneburg M. & Göz G. R. *In vivo* effectiveness of enamel sealants around orthodontic brackets. J Orofac Orthop. 74, 447–457 (2013). 24158583 10.1007/s00056-013-0178-4

[b19] Moriyama C. M., Rodrigues J. A., Lussi A. & Diniz M. B. Effectiveness of fluorescence-based methods to detect *in situ* demineralization and remineralization on smooth surfaces. Caries Res. 48, 507–514 (2014). 24902775 10.1159/000363074

[b20] Claydon N. C. Current concepts in toothbrushing and interdental cleaning. Periodontol 2000 48, 10–22 (2008). 18715352 10.1111/j.1600-0757.2008.00273.x

[b21] Enaia M., Bock N. & Ruf S. White-spot lesions during multibracket appliance treatment: A challenge for clinical excellence. Am J Orthod Dentofacial Orthop. 140, e17–e24 (2011). 21724067 10.1016/j.ajodo.2010.12.016

[b22] Derks A., Kujipens-Jagtman A. M. & Frencken J. E. Caries preventive measures used in orthodontic practices: an evidence-based decision? Am J Orthod Dentofacial Orthop. 132, 165–170 (2007). 17693365 10.1016/j.ajodo.2005.10.028

